# Effect of Solidification on Microstructure and Properties of FeCoNi(AlSi)_0.2_ High-Entropy Alloy Under Strong Static Magnetic Field

**DOI:** 10.3390/e20040275

**Published:** 2018-04-12

**Authors:** Jiaxiang Wang, Jinshan Li, Jun Wang, Fan Bu, Hongchao Kou, Chao Li, Pingxiang Zhang, Eric Beaugnon

**Affiliations:** 1 State Key Laboratory of Solidification Processing, Northwestern Polytechnical University, Xi’an 710072, China; 2 Xi’an Superconducting Magnet Technology Co. Ltd., Xi’an 710018, China; 3 Northwest Institute for Nonferrous Metal Research, Xi’an 710016, China; 4 Laboratoire National des Champs Magnétiques Intenses (LNCMI), University Grenoble Alps, F-38000 Grenoble, France; 5 Laboratoire National des Champs Magnétiques Intenses (LNCMI), French National Center for Scientific Research (CNRS), F-38000 Grenoble, France

**Keywords:** strong static magnetic field, high-entropy alloy, solidification, microstructure evolution, comprehensive properties

## Abstract

Strong static magnetic field (SSMF) is a unique way to regulate the microstructure and improve the properties of materials. FeCoNi(AlSi)_0.2_ alloy is a novel class of soft magnetic materials (SMMs) designed based on high-entropy alloy (HEA) concepts. In this study, a strong static magnetic field is introduced to tune the microstructure, mechanical, electrical and magnetic properties of FeCoNi(AlSi)_0.2_ high-entropy alloy. Results indicate that, with the increasing magnetic field intensity, the Vickers hardness and the saturation magnetization (M_s_) increase firstly, and then decrease and reach the maximum at 5T, while the yield strength, the residual magnetization (M_r_) and the coercivity (H_c_) take the opposite trend. The resistivity values (*ρ*) are found to be enhanced by the increasing magnetic field intensity. The main reasons for the magnetic field on the above effects are interpreted by microstructure evolution (phase species and volume fraction), atomic-level structure and defects (vacancy and dislocation density).

## 1. Introduction

Over the past decades, high-entropy alloys (HEAs) in which their distinctive structures and excellent properties are being continuously explored have attracted extensive attention [1,2,3,4,5,6,7,8,9,10]. HEAs are a novel kind of alloys because they are different from conventional alloys that have one or two principal elements as main components. Yeh et al. firstly propose the concept of HEA and formally define it, which has five or more principal elements in equal or near-equal molar ratios [9]. High configurational entropy that decreases Gibbs free energy in HEAs retards formation of intermetallic compounds and stabilizes single solid solution phases, which include face-centered cubic (FCC), body-centered cubic (BCC), hexagonal cubic phase (HCP), and orthorhombic crystal structures [9,10]. Simple phase species provide convenient conditions for studying the microstructure and properties of HEAs.

With the development of superconducting technologies, the commercial superconducting magnet becomes more easily securable [11]. Strong static magnetic field (SSMF) has aroused extensive interest due to its unique function in materials processing. As a clean, non-contact and high-density energy, the magnetic field can act on the atomic scale of substances. As a result, it brings many unique impacts to the material processing, e.g., levitation [12], nucleation [13], phase transition thermodynamics [14,15], texturing and orientation [16,17,18], organizational refinement [19] and convection [20]. The effect of SSMF on the microstructure during solidification is bound to the properties of the material after solidification, such as mechanical properties [21], thermoelectric properties [21,22] and magnetic properties [23,24]. In the existing literature reports, the role of strong magnetic field in the solidification process focuses on pure metal [20,25], binary alloy system [15,16,17,25] and ternary alloy system [14,18,21,22,23]. Few articles have the application of SSMF during the solidification of HEAs. FeCoNi(AlSi)_0.2_ alloy is a novel class of soft magnetic materials (SMMs) based on HEAs. SMMs require high M_s_, high *ρ*, low H_c_ and low M_r_, in addition to good malleability [26]. Zhang et al. studied the effects of composition changes on microstructure and mechanical, electrical as well as magnetic properties of FeCoNi(AlSi)_x_ HEA [27] Zuo et al. found that the microstructures and magnetic behaviors of FeCoNi(AlSi)_0.2_ HEA were affected by the Bridgman solidification [28]. In this report, SSMF is introduced during the solidification process of FeCoNi(AlSi)_0.2_ HEA, and its effect on the microstructure evolution and properties are studied.

## 2. Experiment and Method

### 2.1. Material Preparation

FeCoNi(AlSi)_0.2_ HEA ingots were prepared by arc-melting under an argon atmosphere. The raw elements were above 99.9 wt %. Each ingot was re-melted four times in order to gain a homogeneous distribution of elements. Then, the ingots were cut into blocks of about 10 g. Finally, each of them was packaged in a quartz tube in a vacuum.

### 2.2. Material Processing Equipment under SSMF

**Figure 1:**
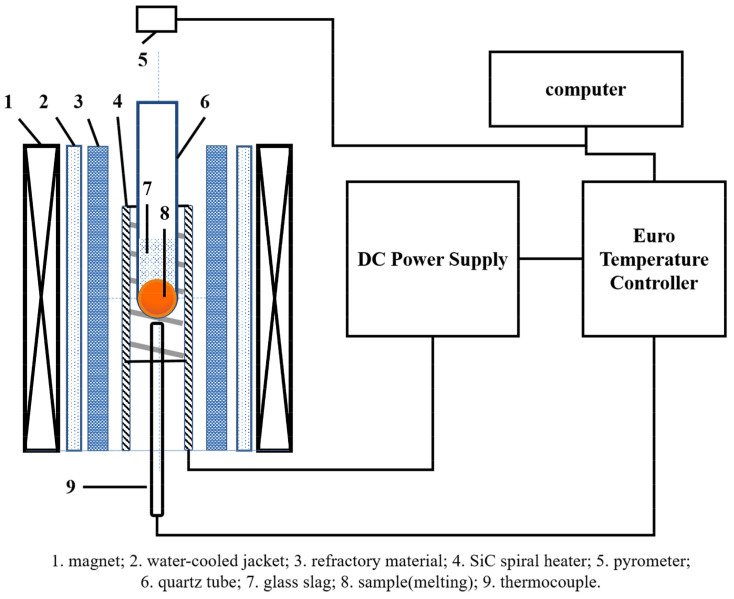
The internal structure and working principle diagram of the solidification equipment under strong static magnetic field.

The experimental setup was a self-built 8-Tesla magnetic field material processing facility. Figure 1 showed the internal structure and working principle diagram of the solidification equipment under SSMF. The sample was placed in the position where the maximum magnetic field intensity and the maximum heating temperature coincided. A two-color pyrometer on top of the equipment was used to in situ measure the change of temperature. The magnetic field intensity was applied to 0T, 1T, 3T, 5T and 7T, respectively. The s-type thermocouple at the bottom of the test tube detected the temperature feedback to the temperature controller, which compared the temperature of the feedback with the temperature of the program to control the power of the power supply. The heating program was firstly heating at the heating rate of 0.3 °C/s, heated to 1450 °C, kept for 1 h, cooled to approximately 1200 °C at a rate of 1 °C/s and quenched in water. 

### 2.3. Analysis and Characterization

**Figure 2:**
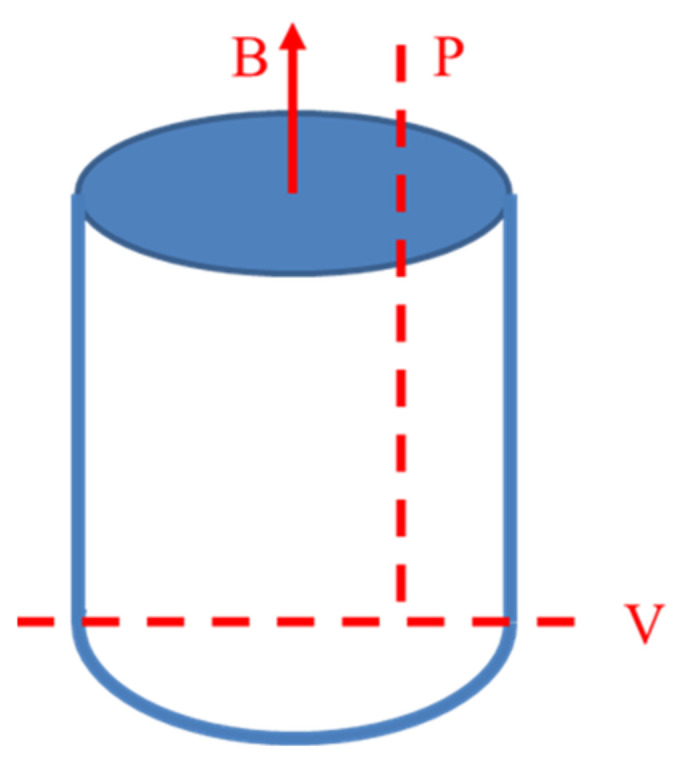
The morphology of the samples solidified under different magnetic fields.

As shown in Figure 2, the morphology of the samples looked like the “bullet”. They were cut along with the direction of “P” and “V”. In the following paper, the “P” and “V” surface represented the direction parallel to the magnetic field and perpendicular to the magnetic field, respectively. The types of crystal structure and phase were detected by X-ray diffractometer (XRD) (Dandong, China, Dandong Haoyuan Instrument Co. Ltd., DX-2700) with a Cu target under radiation from 20° to 100°. The microstructures that were parallel and perpendicular to the magnetic field direction were examined by a ZEISS SUPRA 55 field emission scanning electron microscope (SEM) (Shanghai, China, TESCAN) with the energy-dispersive spectrometry (EDS) (China, TESCAN) and a GX 71 Olympus metallographic microscope (Tokyo, Japan, Olympus). In addition, Ф 2 × 4 mm cylindrical specimens were prepared for compressive experiments at room temperature with a strain rate of 5 × 10^−4^ s^−1^. The hardness was measured by a Vickers hardness tester (St. Joseph, MI, USA, LECO) under a load of 200 g, held for 15 s. The electrical resistivity is tested by a four-probe resistivity tester (Xi’an, China, Xi’an HongHu Testing Instrument Co. Ltd.). The hysteresis loop is tested by a Lake Shore VSM 7307 (Shanghai, China, Shanghai Yihong Scientific Instrument Co. Ltd.) at room temperature.

## 3. Results and Discussion

### 3.1. X-ray Diffraction of FeCoNi(AlSi)_0.2_ Alloy

**Figure 3:**
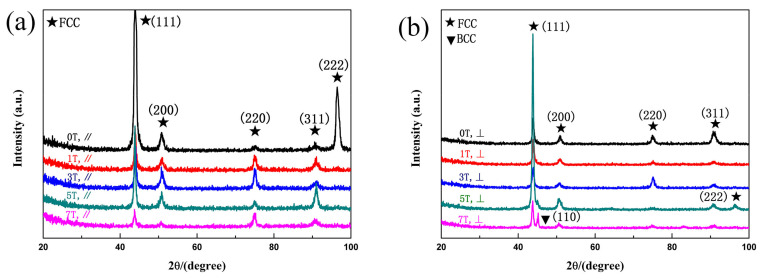
The XRD patterns of FeCoNi(AlSi)_0.2_ HEAs prepared under different magnetic fields: (**a**) //B; (**b**) ⊥B.

Figure 3 shows the XRD patterns of the samples under different magnetic field directions and intensities. The relative strength of the diffraction peaks in the XRD patterns parallel to the magnetic field (Figure 3a) are affected by the magnetic field intensity; however, the specimen is still a single FCC solid solution. Compared with the XRD patterns parallel to the magnetic field, the XRD pattern of the perpendicular direction to the magnetic field (Figure 3b) solidified at 7T reveals a minor peak besides the (111) face-centered-cubic (FCC) peak, which proved to be a body-centered-cubic (BCC) phase. It implies that the process of solidification at 7T can promote the formation of the BCC precipitates. The presence of the BCC phase has a potential impact on the performance of the material below.

### 3.2. Microstructure Characteristics of FeCoNi(AlSi)_0.2_ Alloy

**Figure 4:**
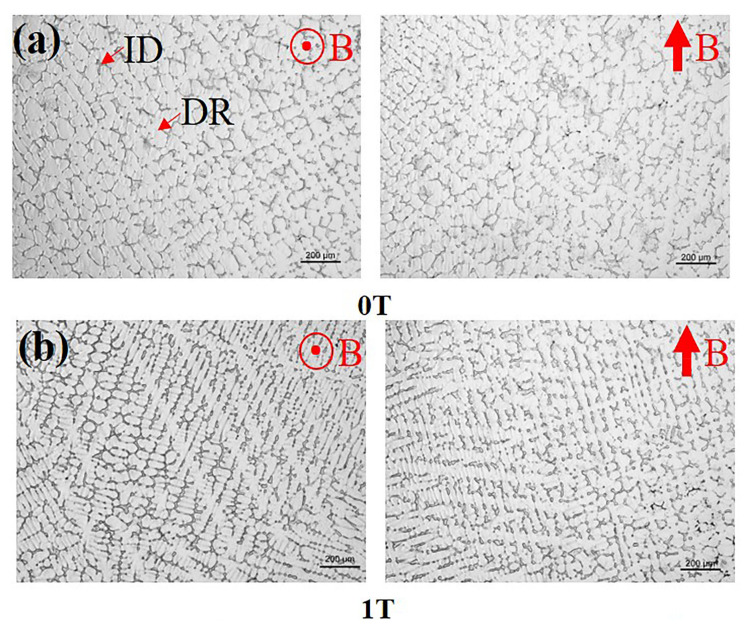

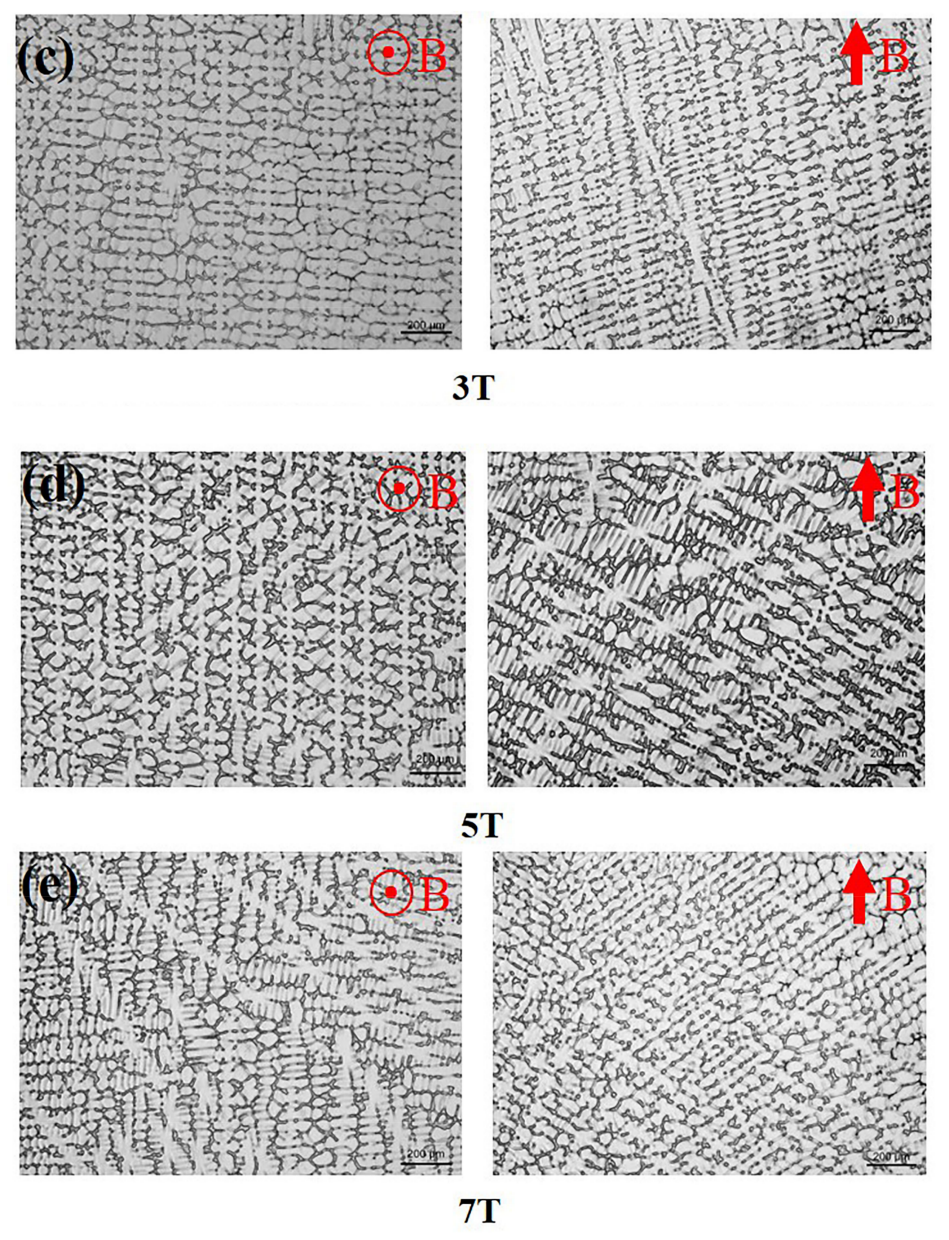
The optical microstructures of FeCoNi(AlSi)_0.2_ HEAs solidified under different magnetic fields: (**a**) 0T; (**b**) 1T; (**c**) 3T; (**d**) 5T; (**e**) 7T.

**Figure 5:**
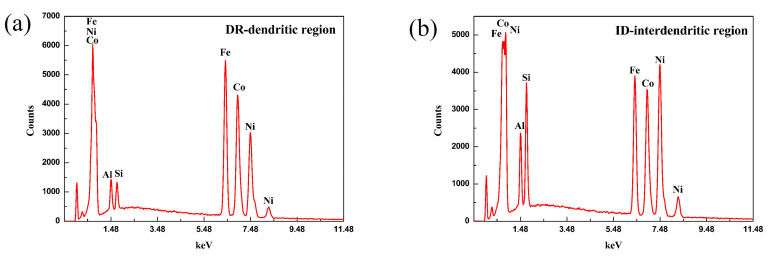
Chemical analysis by the EDS: (**a**) Distribution of elements in the DR region; (**b**) Distribution of elements in the ID region.

**Figure 6:**
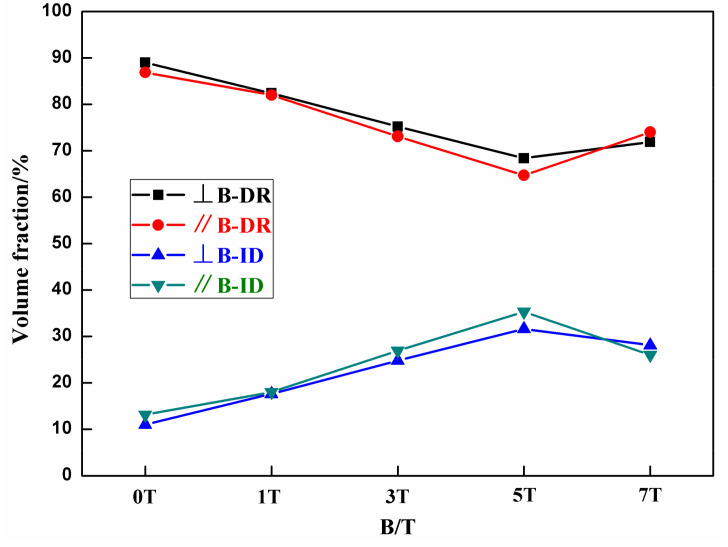
The volume fraction of FeCoNi(AlSi)_0.2_ HEAs under different magnetic fields.

The optical microstructure images of the FeCoNi(AlSi)_0.2_ alloy prepared after solidification under different magnetic field intensities are presented in Figure 4. It is obvious that the microstructure is composed of typical dendritic and interdendritic structures (labeled as “DR” and “ID” by arrows, respectively). Parallel to the magnetic field direction, the primary crystal axis solidified at 0T, 1T, 5T and 7T randomly orientates toward each direction. In particular, orientation of the primary crystal axis solidified at 3T is parallel to the direction of the magnetic field. The EDS results showed in Figure 5 suggest that the Ni, Si and Al in the ID region are rich, while the DR region is rich in Fe and Co. As can be seen from Table 1, the variation of each element atomic percentage of dendrite and interdendritic phase in the different directions of the magnetic field is approximately 1% after solidification under different magnetic field intensities. Therefore, the process of solidification under SSMF has a negligible effect on the diffusion of elements. The change of volume fraction of DR and ID calculated by Image Pro-Plus (IPP) (6.0.0.260) software is shown in Figure 6. With the increasing magnetic field intensity, the volume fraction of DR decreases firstly and then increases. The volume fraction of dendritic phase was the smallest at 5T. The change of volume fraction of ID is correspondingly the opposite. The change in volume fraction is consistent with the mechanical properties in the following sections.

### 3.3. Mechanical Properties of FeCoNi(AlSi)_0.2_ Alloy

**Figure 7:**
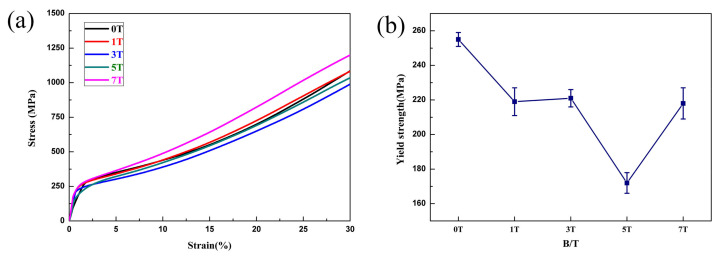
(**a**) The compressive engineering stress–strain curves of FeCoNi(AlSi)_0.2_ HEAs under different magnetic fields (The strain exceeds 50% without fracture); (**b**) The yield strength of FeCoNi(AlSi)_0.2_ HEAs under different magnetic fields.

**Figure 8:**
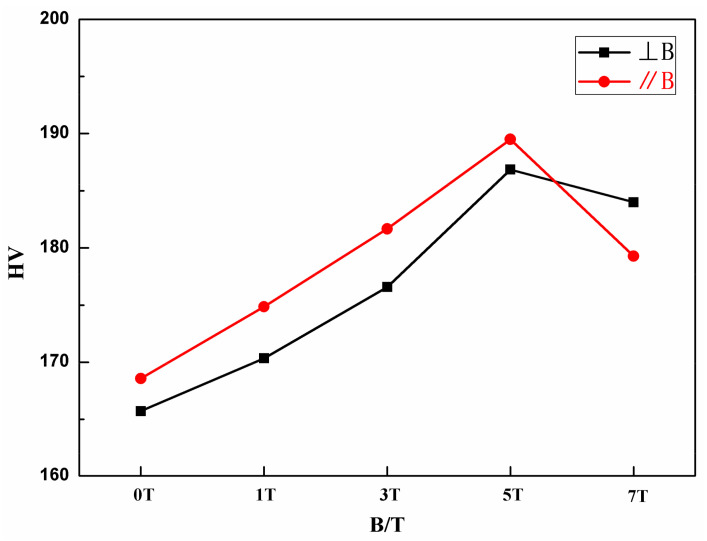
The Vickers hardness of FeCoNi(AlSi)_0.2_ HEAs under different magnetic fields.

The compressive engineering stress–strain curves of the FeCoNi(AlSi)_0.2_ alloys solidified under different magnetic field intensities are shown in Figure 7a. From the stress–strain curves, the alloys solidified under different magnetic field intensities exhibit good plasticity (exceeds 50% without fracture) and significant work hardening. Figure 7b shows that the yield strength decreases firstly and then increases, reaching 172 MPa at 5T. With the increasing magnetic field intensity, the yield strength of FeCoNi(AlSi)_0.2_ alloys show the same trends as the volume fraction of DR. The yield strength at 3T is slightly increased because the microstructure orientation at 3T is parallel to the direction of the magnetic field. In addition, the direction of the pressure applied in the compression test is along the direction of the magnetic field. Figure 8 shows the Vickers hardness values of the FeCoNi(AlSi)_0.2_ alloys solidified under SSMF. With the increasing magnetic field intensity, the Vickers hardness of FeCoNi(AlSi)_0.2_ alloys show the same trends as the volume fraction of ID. The Vickers hardness values increase firstly and then decrease, the maximum value is 190 HV and 187 HV at 5T in the parallel or perpendicular direction of the magnetic field, respectively.

### 3.4. Electrical Resistivity of FeCoNi(AlSi)_0.2_ Alloy

**Figure 9:**
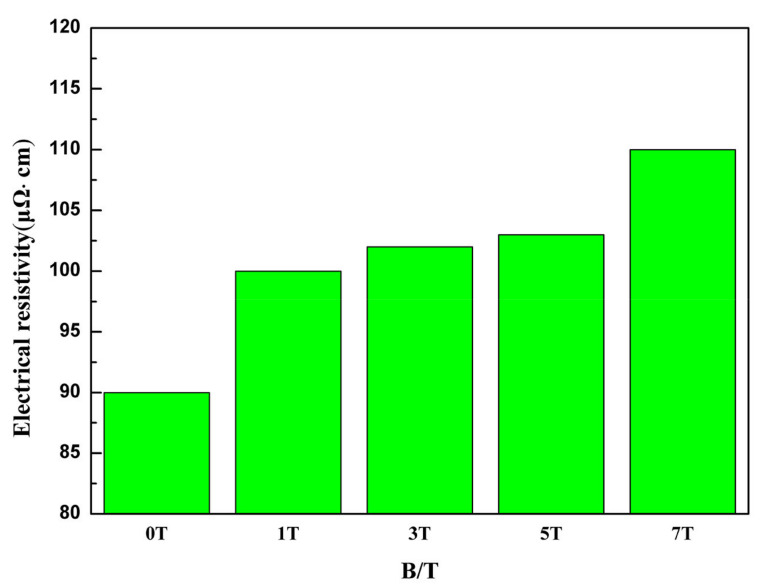
The electrical resistivity of FeCoNi(AlSi)_0.2_ HEAs under different magnetic fields.

Figure 9 shows the electrical resistivity values of the specimen under SSMF tested by using the four-probe method. A small cylinder with a thickness of 3 mm is intersected on the sample after solidification under different magnetic field intensities. In addition, the axis of the small cylinder is parallel to the magnetic field direction. The calculation formula is below:

(1) $$\rho_{V}=R_{V}\frac{S}{h}\;,$$

where R_V_ is resistance, S is the area of the electrode and h represents thickness of specimen (i.e., distance between two electrodes). The electrical resistivity of the silicon steel is 50–80 μΩ·cm [29]. The as-cast FeCoNi(AlSi)_0.2_ is 69.5 μΩ·cm [27]. Compared with the resistivity of the as-cast sample, the resistivity of the sample after solidification under different magnetic field intensities is greatly improved. This is because the solidification under SSMF will experience the process of high temperature quenching and rapid cooling in water, which will cause the defects of the metal to be far beyond the equilibrium concentration. In this experiment, the quenching temperature is close to the melting point, and the residual resistivity caused by the vacancy of the quenching “frozen” is shown below [30]:

(2) $$\Delta \rho ={\mathrm{Ae}}^{-\frac{E}{\mathrm{kT}}}\;,$$

where E is the formation energy of vacancy, T is quenching temperature, A and k are constants. The influence of vacancy on residual resistivity is similar to that of impurity atom in metal, and their effect size is the same order of magnitude. Tian et al. found the influence of vacancy and dislocation on the residual resistivity in some pure metals, such as copper, silver, platinum, iron and so on [30]. With the increasing magnetic field intensity, the resistivity has always been increased. Wang et al. found that the magnetic field can induce an increase of dislocation density [19]. In addition, it is speculated that the change of resistivity can be caused by the increase of dislocation density due to the magnetic field. The discontinuous change at 7T may have some relation to the precipitated BCC phase. They still need to be verified by further experiments. High electrical resistivity can reduce the eddy-current loss, which is a wonderful requirement for SMMs particularly used in the high-frequency magnetic field [29]. Therefore, the solidification under different magnetic fields can improve the soft magnetic properties of FeCoNi(AlSi)_0.2_ HEA.

### 3.5. Magnetic Properties of FeCoNi(AlSi)_0.2_ Alloy

**Figure 10:**
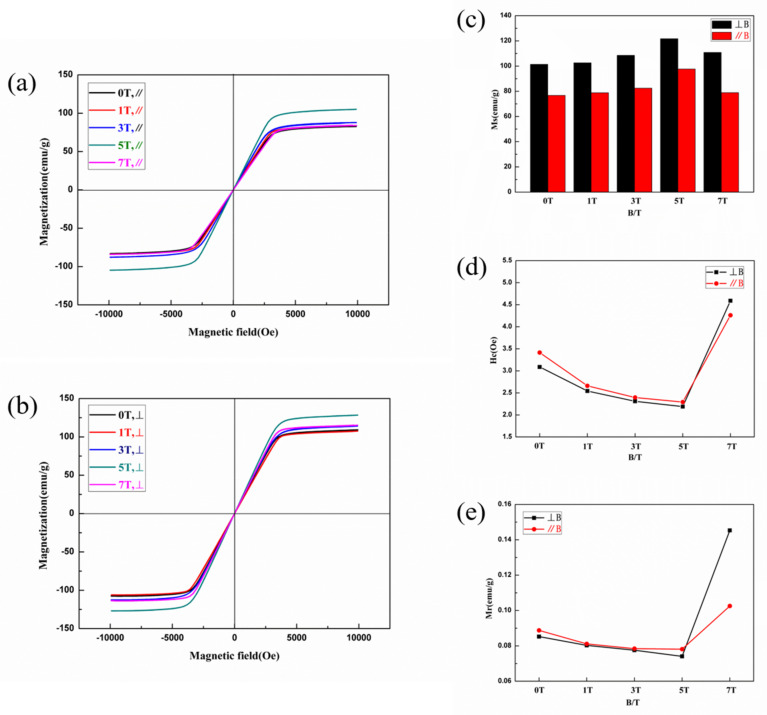
Magnetic hysteresis loops of FeCoNi(AlSi)_0.2_ HEAs: (**a**) //B; (**b**) ⊥B; (**c**) the variation tendency of M_s_; (**d**) the variation tendency of H_c_; (**e**) the variation tendency of M_r_.

Magnetic properties of the samples solidified under SSMF are plotted in Figure 10. The maximum M_s_ of the vertical and parallel magnetic field direction is 121.7 emu/g and 97.7 emu/g at 5T, respectively. H_c_ also reaches its minimum value at 5T regardless of magnetic field direction. The corresponding values are 2.19 Oe and 2.29 Oe. In addition, the variation tendency of M_r_ is the same as that of H_c_. M_s_ is mainly influenced by composition and atomic-level structures, and less sensitive to microstructures, such as the grain size and morphology [31]. In this experiment, the composition is the same, and the microstructure solidified under different magnetic field intensities is basically unchanged. The variation of M_s_ may be attributed to the influence of the magnetic field on the atomic-level structure of FeCoNi(AlSi)_0.2_ HEA. However, the specific influence mechanism still needs to be studied further. Different from M_s_, H_c_ will be significantly affected by defects, grain size, heat treatment processes and so on [31]. It is apparent that the grain boundary impedes the movement of the domain wall. Therefore, larger grain size and lower grain boundary density have the lower H_c_. The equation of H_c_ is shown below [32,33]:

(3) $$H_{c}\approx 3\sqrt{\frac{K_{B}T_{c}K_{1}}{{\alpha M}_{s}}}\frac{1}{D}\;,$$

where H_c_ is the coercivity, D the grain size, M_s_ the magnetization saturation, K_1_ the magneto-crystalline anisotropy, K_B_ the Boltzmann constant, T_c_ the Curie temperature, and α the lattice constant. When the grain size is almost the same, the change trend of H_c_ may be related to M_s_ and K_1_ from this equation. When M_s_ reaches the maximum, the coercivity has the minimum value. In addition, the abnormal change of magnetic properties (M_s_, H_c_ and M_r_) at 7T may be related to the precipitated BCC phase. In the SMMs, high M_s_, low H_c_ and low M_r_ are excellent results for use. In short, the magnetic behaviors of FeCoNi(AlSi)_0.2_ HEA are improved by the solidification under the magnetic field, and the soft magnetic properties of the alloy are further optimized.

## 4. Conclusions

In this study, the microstructure evolution and properties of FeCoNi(AlSi)_0.2_ HEA solidified under SSMF have been systematically investigated. We find that the solidification technology under magnetic field can be used to improve the soft magnetic properties of the alloy. The main conclusions are as follows:

(1) During the solidification of different magnetic field intensities, the alloy solidified up to 5T is still a single FCC phase, and has the maximum Vickers hardness values (190 HV and 187 HV in the parallel and perpendicular direction of the magnetic field, respectively) and the minimum yield strength value (172 MPa).
(2) The electrical resistivity increases with the magnetic field intensity, and the maximum value is 110 μΩ·cm at 7T.
(3) The maximum saturation magnetization values of the vertical and parallel magnetic field direction are 121.7 emu/g and 97.7 emu/g at 5T. The minimum coercivity values are 2.19 Oe and 2.29 Oe at 5T, respectively.

## Figures and Tables

**Table 1:** Chemical analysis of FeCoNi(AlSi)_0.2_ HEAs at different regions or different magnetic fields.

B		Area	Al	Si	Fe	Co.	Ni
As-cast		DR	6.063	5.300	31.207	30.253	27.177
		ID	9.963	14.770	20.830	23.050	31.387
0T	⊥	DR	6.370	4.740	31.760	30.417	26.710
		ID	10.193	14.573	19.830	22.050	33.353
	//	DR	6.467	4.793	31.910	20.283	26.547
		ID	10.987	15.200	19.733	21.677	32.400
1T	⊥	DR	6.407	4.707	32.037	30.533	26.317
		ID	10.923	15.043	19.487	21.730	32.823
	//	DR	6.800	4.800	31.583	30.343	26.470
		ID	11.217	14.957	19.413	21.543	32.870
3T	⊥	DR	6.677	4.623	32.013	30.390	26.297
		ID	11.077	15.327	19.617	21.640	32.333
	//	DR	6.723	5.483	30.930	30.160	26.707
		ID	11.317	15.873	19.173	21.397	32.240
5T	⊥	DR	6.380	4.973	31.140	30.423	27.083
		ID	9.837	14.600	20.050	22.243	33.267
	//	DR	6.343	4.873	31.857	30.343	26.570
		ID	10.597	14.423	19.803	22.113	33.057
7T	⊥	DR	6.807	5.027	31.630	30.137	26.400
		ID	11.046	14.887	19.713	21.467	32.887
	//	DR	6.807	5.027	31.630	30.137	26.400
		ID	11.047	14.887	19.713	21.467	32.887
